# Identification of G6PC as a potential prognostic biomarker in hepatocellular carcinoma based on bioinformatics analysis

**DOI:** 10.1097/MD.0000000000029548

**Published:** 2022-08-19

**Authors:** Li Tian, Yong Liao

**Affiliations:** a Key Laboratory of Molecular Biology on Infectious Diseases, Ministry of Education, Chongqing, China; b Institute for Viral Hepatitis, Chongqing Medical University, Chongqing, China; c Department of Infectious Diseases, Second Affiliated Hospital, Chongqing Medical University, Chongqing, China.

**Keywords:** bioinformatics, differentially expressed genes, glucose-6-phosphatase catalytic, hepatocellular carcinoma, prognosis

## Abstract

Hepatocellular carcinoma (HCC) has high mortality and incidence rates around the world with limited therapeutic options. There is an urgent need for identification of novel therapeutic targets and biomarkers for early diagnosis and predicting patient survival with HCC.

Several studies (GSE102083, GSE29722, GSE101685, and GSE112790) from the GEO database in HCC were screened and analyzed by GEO2R, gene ontology and Kyoto Encyclopedia of Genes and Genomes enrichment analysis were conducted with the Database for Annotation, Visualization and Integrated Discovery. The protein-protein interaction network was plotted and the module analysis was performed using Search Tool for the Retrieval of Inter-acting Genes/Proteins database and Cytoscape. The expression and survival of key genes were identified using UALCAN, Kaplan–Meier Plotter and ONCOMINE online databases, and the immune infiltration level of key genes was analyzed via the Tumor Immune Estimation Resource (TIMER) database.

Through database analysis, eight key genes were finally screened out, and the expressions of cyclin-dependent kinase regulatory subunit 2 and glucose-6-phosphatase catalytic (G6PC), which were closely related to the survival of HCC patients, was detected by using UALCAN. Further analysis on the differential expression of G6PC in multiple cancerous tumors and normal tissues revealed low expression in many solid tumors by Oncomine and TIMER. In addition, Kaplan–Meier plotter and UALCAN database analysis to access diseases prognosis suggested that low expression of G6PC was significantly associated with poor overall survival in HCC patients. Finally, TIMER database analysis showed a significant negative correlation between G6PC and infiltration levels of six kinds of immune cells. The somatic copy number alterations of G6PC were associated with B cells, CD8^+^ T cells, CD4^+^ T cells, macrophages, dentritic cells and neutrophils.

These bioinformatic data identified G6PC as a potential key gene in the diagnosis and prognosis of HCC.

## 1. Introduction

The latest 2020 global cancer statistics report released by the International Agency for Research on Cancer of the World Health Organization showed that live cancer is a major global health problem that both developing and developed countries are facing.^[1]^ Hepatocellular carcinoma (HCC) is the most common liver cancer, accounting for approximately 90% of cases.^[2]^ Local regional metastasis and recurrence are the primary causes of death in patients with HCC.^[3]^ At present, although there are a variety of treatments for HCC, such as hepatectomy, liver transplantation and molecular targeting drugs (sorafenib), the treatment effect is still not ideal due to high recurrence and high metastasis of HCC.^[4–6]^ Besides, the 5-year overall survival rate of advanced HCC is still less than 18% and the main causes of poor prognosis were tumor metastasis and postoperative recurrence.^[7–9]^ However, most patients were in the stage of advanced HCC by the time of diagnosis in the clinic, and lost the opportunity of surgical resection.^[10–12]^ Therefore, screening out new potential biomarkers for HCC prognosis is of great significance for reducing the mortality of HCC patients, improving prognosis and realizing individualized targeted therapy.

With the rapid development of high-throughput sequencing technology and bioinformatics technology, more and more researchers are using bioinformatics analysis to explore the molecules and pathways that play an important role in the occurrence and development of HCC so as to predict potential biomarkers of HCC.^[13,14]^ Gene Expression Omnibus (GEO) provides us with a lot of disease-related expression profile information, which contains more than 32,000 public datasets from 13,000 laboratories, including second-generation gene chip sequencing and high-throughput sequencing, providing important data support for multi-sample tumor studies.^[15,16]^ More and more studies are based on data mining of GEO platform, and a large number of experiments have proved that the key genes mined play an important role in the process of cancer occurrence and development.^[17,18]^ This study used the GEO platform dataset and aimed to integrate multiple datasets to find biomarkers related to HCC prognosis. Gene chip is an efficient and high-throughput technology to obtain biological information, which can detect and analyze differentially expressed genes (DEGs) between HCC and normal liver tissues.^[19,20]^ In this study, four HCC gene chips were downloaded from GEO database, including GSE102083, GSE29722, GSE101685 and GSE112790. GEO2R was used to identify DEGs in 337 HCC tissues and normal tissues. Next, the Database for Annotation, Visualization and Integrated Discovery (DAVID) database was applied to carry out the gene ontology (GO) Functional Annotation and Kyoto Encyclopedia of Genes and Genomes (KEGG) Pathway analysis. The Protein-protein interaction (PPI) network was built by using Search Tool for the Retrieval of Interacting Genes (STRING) and visualized with Cytoscape. The gene with the highest Molecular Complex Detection (MCODE) score in each module was taken as the key gene. Among the identified genes, the expression of cyclin-dependent kinase regulatory subunit 2 (CKS2) and glucose-6-phosphatase catalytic (G6PC) was closely related to poor prognosis. Besides, G6PC in HCC is rarely reported. Therefore, we used several databases such as UALCAN, Kaplan–Meier plotter and ONCOMINE to comprehensively analyze G6PC expression and its association with prognosis. In addition, Tumor Immune Estimation Resource (TIMER) analysis displayed the expression of G6PC and correlation with tumor-infiltrating immune cells. These results provided strong evidence to illustrate that G6PC could be a potential biomarker to predict HCC diagnosis and prognosis.

## 2. Materials and Methods

### 2.1. Screening microarray datasets

GEO database (https://www.ncbi.nlm.nih.gov/geo/)^[21]^ stores data from second generation chips and we can retrieve some experimental sequencing data uploaded by others. We downloaded four microarray datasets (GSE102083, GSE29722, GSE101685, and GSE112790) from GEO datasets. They have the following four characteristics in common: (a) Expression profiling by array; (b) The sample was composed of HCC and normal liver samples; (c) They all came from the same platform: GPL570 [HG-U133_Plus_2] Affymetrix Human Genome U133 Plus 2.0 Array; (d) They were updated recently (2019–2020). These datasets contained 369 HCC and 47 liver samples altogether. GSE102083 included 152 liver cancers and 14 normal livers in Japan. GSE29722 contained 10 pairs of tumor samples and normal liver tissues in Canada. GSE101685 included 24 liver cancer samples and 8 normal liver controls in Taiwan and GSE112790 contained 183 HCC tissues and 15 liver tissues in Japan.

### 2.2. Identification of DEGs

GEO2R (https://www.ncbi.nlm.nih.gov/geo/geo2r/)^[21]^ can help us to analyze DEGs, which is an useful online software. Setting adjusted *P* < .05 and LogFC (Fold Change) > 1.5 or <−1.5 to define DEGs. LogFC < −1.5 was considered upregulated genes and LogFC > 1.5 down-regulated genes. An online tool − Calculate and draw custom Venn diagrams (http://bioinformatics.psb.ugent.be/webtools/Venn/) was used to calculate the overlap of DEGs.

### 2.3. Functional enrichment analysis of DEGs

The DAVID (https://david.ncifcrf.gov/)^[22–24]^ database was employed to carry out Gene Ontology functional and Kyoto Encyclopedia of Genes and Genomes pathway analysis of DEGs, GO terms contain biological process (BP), cellular component and molecular function. KEGG^[25]^ pathway analysis is helpful for further understanding the function of DEGs. The screening criteria were *P*<.01 with gene counts >10.

### 2.4. PPI Network Construction and module analysis

STRING/Proteins database (https://string-db.org/)^[26]^ is a database that searches for protein-protein interactions. We selected “Multiple proteins” in the left column and entered gene names in the right column, and then picked the organism as “Homo sapiens”, choose the minimum required interaction score as “medium confidence (0.400)” and hided disconnected nodes in the network, clicked the “Export” option, downloaded the file in TSV format and imported it into Cytoscape software (version 3.7.2; https://cytoscape.org/)^[27]^ which is a very powerful tool for visualizing network data. Then, a plug-in in Cytoscape MCODE^[28]^ was used to cluster the protein network to build functional modules. The default parameters are: degree cutoff = 2, node density cutoff = 0.1, node score cutoff = 0.2, k-core = 2 and max.depth = 100.

### 2.5. Expression and survival analysis of hub genes

In order to comprehensively analyze the effect of key gene expression on survival rate, we used the following databases. UALCAN (http://ualcan.path.uab.edu./) is an online website that can be used to mine and analyze cancer data, and the main source of the database is The Cancer Genome Atlas (TCGA), including expression profiling and survival analysis.^[29]^ First, we entered gene symbols, selected TCGA dataset, and then clicked “Expression” or “Survival” to obtain the expression level of hub genes and its effect on patient survival. *P* value was less than .01.

Two genes that met the above screening criteria − CKS2 and G6PC. Kaplan–Meier Plotter (http://kmplot.com/analysis/) was used to evaluate the overall survival rate of key genes.^[30]^ Moreover, we also performed CKS2 and G6PC correlation analysis via Gene Expression Profiling Interactive Analysis (GEPIA; http://gepia.cancer-pku.cn/).^[31]^ The threshold was logrank *P* value <.01 in Kaplan–Meier Plotter and *P* value <.01 in GEPIA. Literature review showed that CKS2 has been proved to predict the prognosis of HCC, so next G6PC was selected to conduct further analysis. ONCOMINE (https://www.oncomine.org/) is a tumor database which can compare gene expression between normal and cancerous tissues in different cancers.^[32]^ The threshold was *P* value <.01, fold change <1.5, and gene rank came from all. A stratified analysis of G6PC was also conducted according to patients’ gender, race and pathological stage by Kaplan–Meier Plotter (log rank *P* < .05).

### 2.6. TIMER database analysis

TIMER (https://cistrome.shinyapps.io/timer/) website, which is divided into seven modules, the first six modules present TCGA data and some analysis, and the last module provides quantitative analysis of the infiltration level of immune cells.^[33,34]^ Here, we chose Diff Exp module to study the differential gene expression between tumor and normal tissues; Gene module to visualize the correlation of its expression with immune infiltration level in diverse cancer types and somatic copy number alteration (SCNA) module to compare infiltration levels among tumors with different SCNAs. SCNAs include deep deletion, arm-level deletion, diploid/normal, arm-level gain, and high amplification. *P* value less than .01 was considered statistically significant.

## 3. Results

### 3.1. Screening and identification of DEGs in HCC

**Figure 1.**
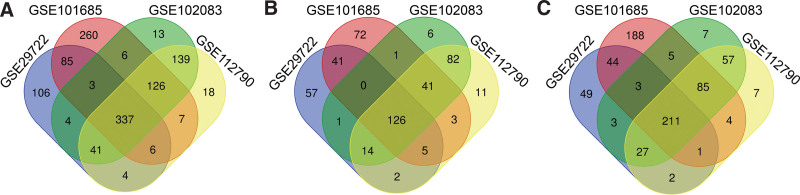
Identification of common DEGs in mRNA expression profiling datasets through Venn diagram analysis. (A) An overlap of 337 DEGs from these four datasets were selected with adjusted *P* < .05 and |logFC| > 1.5. (B) Overlapping upregulated DEGs. (C) Overlapping downregulated DEGs. Abbreviation: DEGs = differently expressed genes.

Details of gene expression profile datasets such as dataset ID, country, the number of tumor and normal samples, and platform information, were demonstrated in Table 1. Totally, 669 DEGs (271 upregulated genes and 398 down-regulated genes), 586 DEGs (246 upregulated genes and 340 down-regulated genes), 830 DEGs (289 upregulated genes and 541 down-regulated genes) and 678 DEGs (284 upregulated genes and 394 down-regulated genes) in GSE102083, GSE29722, GSE101685, and GSE112790 datasets, respectively. Venn diagram online tool was used to calculate overlapped DEGs. A sum of 337 common DEGs (Fig. 1A) was found in all four datasets, consisting of 126 upregulated genes (Fig. 1B) and 211 down-regulated genes (Fig. 1C).

**Table 1:** Details of four gene chip datasets.

Datasets ID	Country	Platform	Tumor samples	Normal samples
GSE102083	Japan	GPL570	152	14
GSE29722	Canada	GPL570	10	10
GSE101685	Taiwan	GPL570	24	8
GSE112790	Japan	GPL570	183	15

GPL = gene expression omnibus platform, GSE = gene expression omnibus series.

### 3.2. GO and KEGG pathway analysis of DEGs

**Figure 2.**
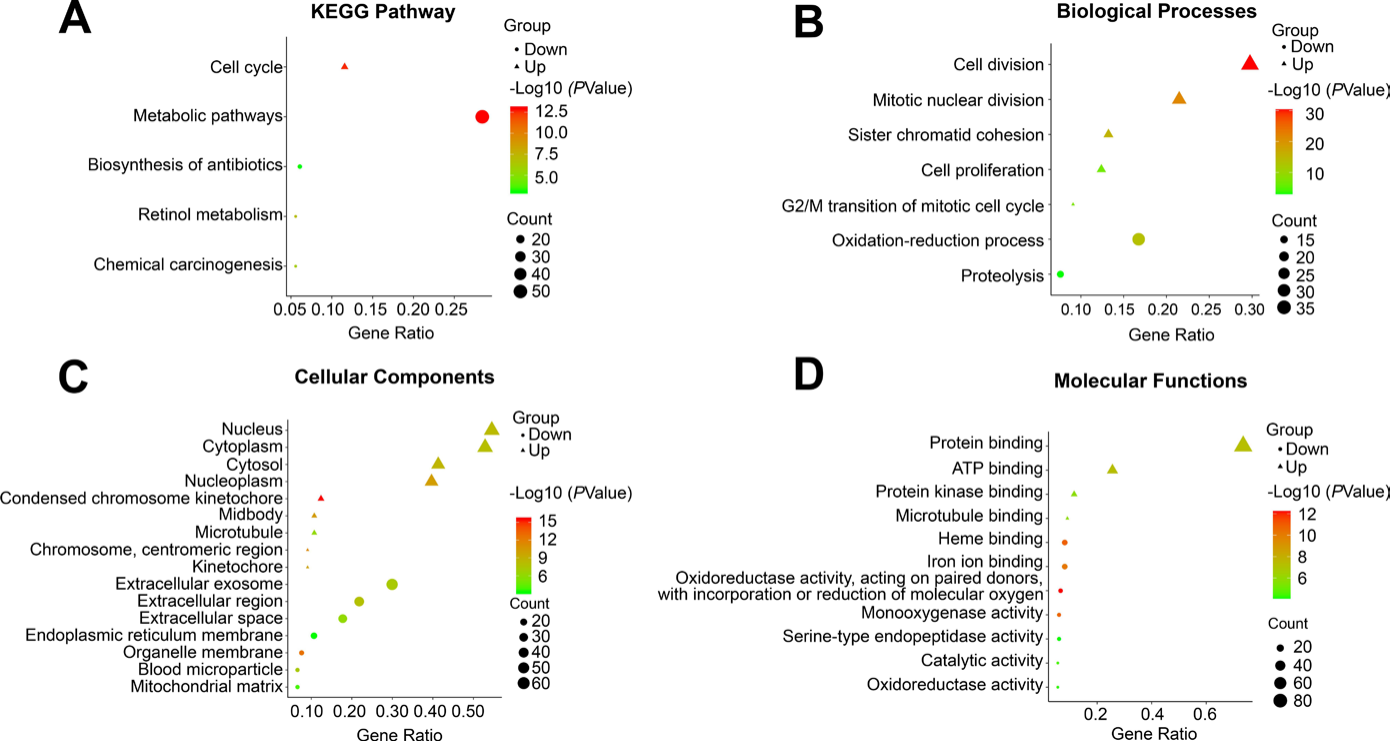
Functional enrichment analysis of the overlap DEGs. (A) The KEGG pathway enrichment analysis of DEGs. (B) Enrichment of biological processes. (C) Enrichment of cellular components. (D) Enrichment of molecular functions. The screening criteria was *P* < .01 with gene counts >10. Color variable represents −Log10 (*P* value), point size variable represents gene count, shape variable represents different groups, triangle represents upregulated genes, circle represents downregulated genes. Abbreviations: DEGs = differently expressed genes, Down = downregulated genes, KEGG = Kyoto encyclopedia of genes and genomes, Up = upregulated genes.

GO and KEGG pathway analysis was performed by DAVID. The results of KEGG showed that upregulated genes were mainly enriched in cell cycle, while down-regulated genes were significantly involved in metabolic pathways and retinol metabolism (Fig. 2A).

For upregulated genes, the BP included cell division and mitotic nuclear division. For down-regulated genes, the BP contained oxidation-reduction process and proteolysis (Fig. 2B). In terms of cellular components, upregulated genes were most accumulated in condensed chromosome kinetochore and chromosome, centromeric region, while down-regulated genes in organelle membrane and extracellular region (Fig. 2C). In addition, molecular function analysis indicated that upregulated genes predominantly participated in Adenosine Triphosphate (ATP) binding and protein binding, while down-regulated genes in oxidoreductase activity, acting on paired donors, with incorporation or reduction of molecular oxygen and heme binding (Fig. 2D).

### 3.3. Modular analysis and hub gene identification

**Figure 3.**
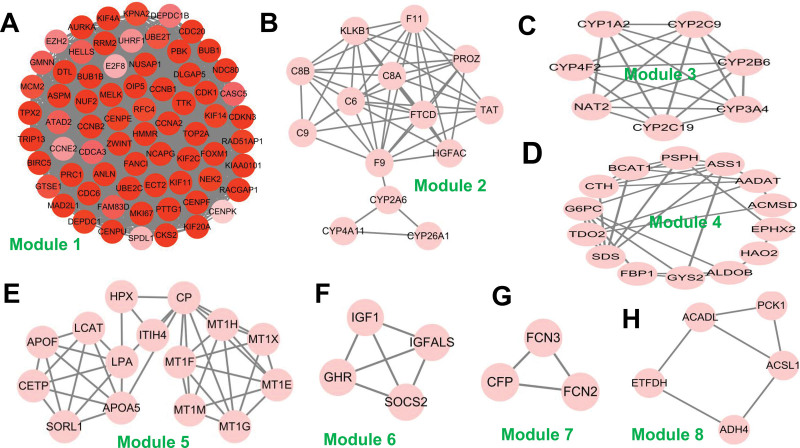
PPI network and MCODE modular analysis of DEGs. The PPI network of DEGs was constructed using Cytoscape software and divided into 8 modules via MCODE plugin. The node color from light to dark represents the MCODE score from low to high. (A–H) stands for module 1–module 8. Abbreviations: DEGs = differently expressed genes, MCODE = molecular complex detection, PPI = protein-protein interaction.

The PPI networks of 337 common DEGs were constructed using the STRING website and then imported into Cytoscape to identify critical gene modules. It demonstrates that a total of 309 items were filtered into the PPI network, and 37 of the 309 DEGs were disconnected from the network. Ultimately, a total of 272 nodes and 3110 edges were filtered by Cytoscape software. Then we analyzed the entire PPI network using MCODE plugin and obtained 8 modules (Fig. 3). Genes with the highest MCODE score of each module were selected as the hub genes. The node color from light to dark indicates the MCODE scores are from low to high. Table 2 presented the network information related to hub genes, including degree, betweenness centrality and closeness centrality.

**Table 2:** Hub gene related network information.

Genes	MOCDE Score	Degree	Betweenness centrality	Closeness centrality
CKS2	50.78616352	59	0.00048200	0.88311688
C8B	7	8	0.0042735	0.65
CYP3A4	5.333333333	6	0.03333333	1
LPA	5	7	0.23260073	0.66666667
G6PC	4	6	0.22649573	0.61904762
SOCS2	3	3	0	1
CFP	3.047619048	2	0	1
ACADL	3	3	0.25	0.8

ACADL = Acyl-CoA dehydrogenase long chain, C8B = complement C8 beta chain, CFP = complement factor properdin, CKS2 = cyclin-dependent kinase regulatory subunit 2, CYP3A4 = cytochrome P450 family 3 subfamily A member 4, G6PC = glucose-6-phosphatase catalytic, LPA = lipoprotein(A), MCODE = molecular complex detection, SOCS2 = suppressor of cytokine signaling 2.

Validation of G6PC and CKS2 expression and survival analysis in a variety of cancers.

**Figure 4.**
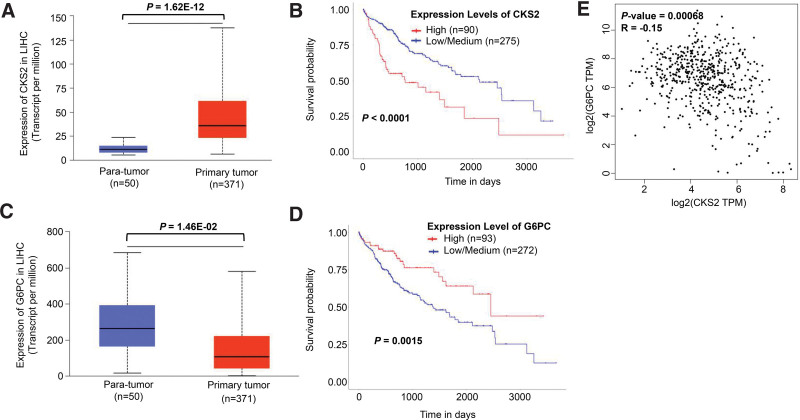
Alterations of hub gene expression and the impact on patients survival. CKS2 was highly expressed in HCC than that in normal tissues and its high expression had relation with unfavorable prognosis (A, B). While G6PC was lowly expressed in HCC compared with normal samples and its low expression had an adverse effect on patients survival (C, D). There was a connection between CKS2 expression and G6PC expression (E). Abbreviations: CKS2 = cyclin-dependent kinase regulatory subunit 2, HCC = hepatocellular carcinoma, G6PC = glucose-6-phosphatase catalytic.

**Figure 5.**
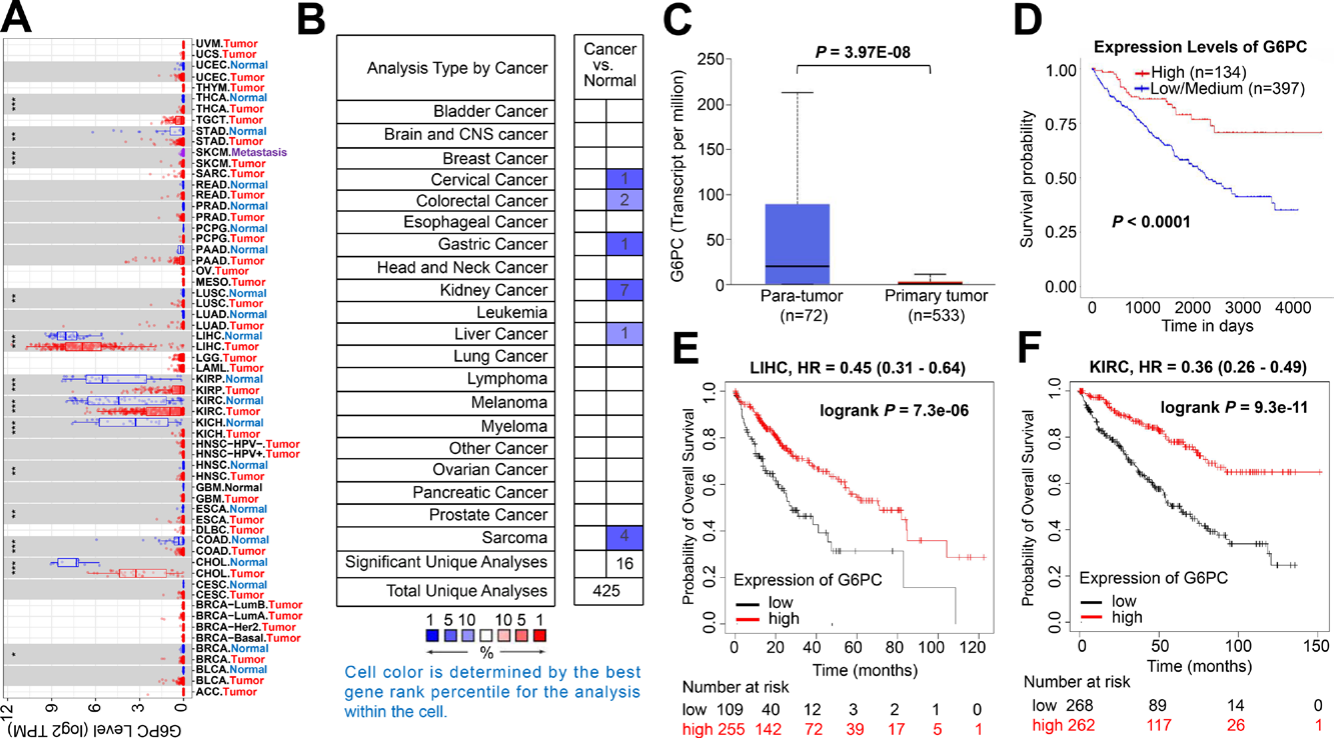
G6PC expression in multiple cancers and its influence on OS. TIMER and ONCOMINE results both illustrated that G6PC was lowly expressed in COAD, STAD, LIHC, and KIPAN (^*^P < .05, ^**^P < .01, ^***^P < .001, A, B). We then employed UALCAN to determine whether G6PC expression had correlation with patients survival in several cancers above except for HCC (*P* < .01). Results showed that G6PC was expressed lowly in KIRC as well and its low expression was associated with poor survival (C, D). Kaplan–Meier Plotter confirmed that G6PC expression was in connection with poor OS in HCC and KIRC again (E, F). Abbreviations: COAD = colon adenocarcinoma, G6PC = glucose-6-phosphatase catalytic, HCC = hepatocellular carcinoma, KIPAN includes KICH (kidney chromophobe), KIRC (kidney renal clear cell carcinoma) and KIRP (kidney renal papillary cell carcinoma)LIHC = liver hepatocellular carcinoma, OS = overall survival, STAD = stomach adenocarcinoma, TIMER = tumor immune estimation resource.

The UALCAN platform was applied to validate the expression level and its effect on survival rate. We verified 8 key genes by putting them on the UALCAN website and found that the expression level of CKS2 in tumor tissues was higher than that in normal tissues (*P* = 1.62E-12). The levels of CKS2 expression were inversely correlated with survival in HCC patients, that is, the higher the expression of CKS2, the lower the survival rate was (*P* < .0001, Fig. 4A, B). Whereas, G6PC had lower expression in tumor specimens compared to normal or para-tumor specimens (*P* = 1.46E-02), and the lower level of G6PC expression the lower probability of a long-term survival in patients with HCC (*P* = .0015, Fig. 4C, D). We also performed GEPIA to assess the correlation between CKS2 and G6PC, and found that there was a correlation between CKS2 expression and G6PC expression (*P* = .00068, *R* = −0.15, Fig. 4E). In addition, CKS2 has already been reported as a prognostic indicator in HCC, and G6PC has been validated as a prognostic biomarker in KIRC.^[35,36]^ Hence, we chose G6PC instead of CKS2 for further verification in HCC and other tumor types. We used ONCOMINE and TIMER to study the expression of G6PC across various TCGA tumors. We found that COAD (Colon adenocarcinoma), STAD (Stomach adenocarcinoma), liver hepatocellular carcinoma, and KIPAN had lower G6PC expression in both TIMER (Fig. 5A) and ONCOMINE databases (Fig. 5B, ^**^P < .01, ^***^P < .001). KIPAN includes KICH (Kidney chromophobe), KIRC (Kidney renal clear cell carcinoma), and KIRP (Kidney renal papillary cell carcinoma). We then used UALCAN to evaluate the impact of G6PC expression on patients survival in these types of cancers and the result demonstrated that G6PC had lower expression in KIRC in comparison with normal specimens (*P* = 3.97E-08), and the lower expression level was related to poor survival rate (*P* < .0001, Fig. 5C, D). Meanwhile, Kaplan–Meier Plotter analysis demonstrates that the lower levels of G6PC expression, the poorer prognosis in patients with either liver hepatocellular carcinoma (log rank *P* = 7.3E-06) or KIRC (log rank *P* = 9.3E-11, Fig. 5E, F).

### 3.4. G6PC expression predicts OS (overall survival) in patients with HCC

**Figure 6.**
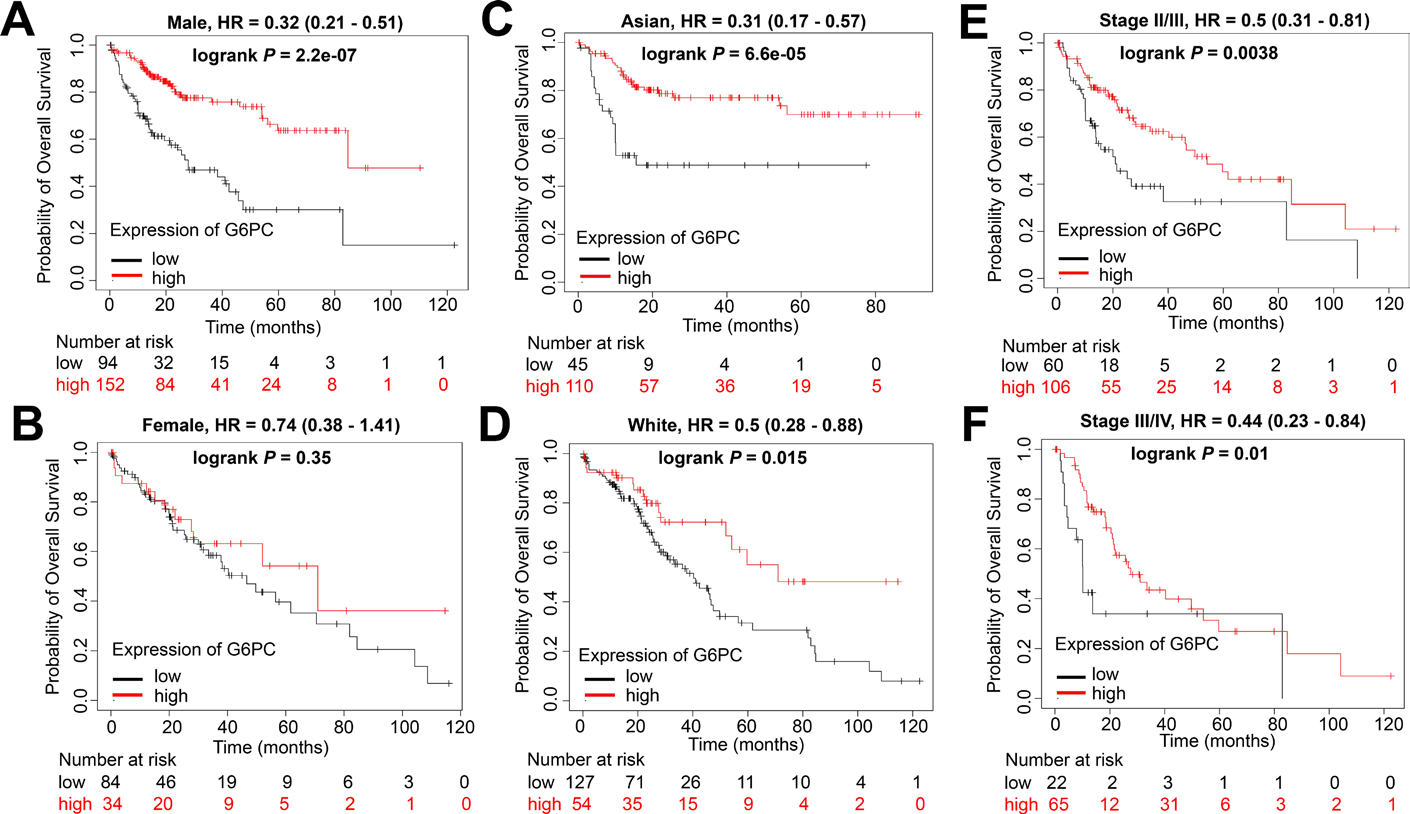
G6PC expression predicts HCC patients OS independent on race and stage, but gender (log rank *P* < .05). There were significant differences in the expression of G6PC between male (A) and female (B) patients. No significant differences in G6PC expression were found between Asian (C) and White (D) HCC patients. There were no significant differences in G6PC expression between stage 2 to 3 (E) and stage 3 to 4 (F) HCC patients as well. Abbreviations: G6PC = glucose-6-phosphatase catalytic, HCC = hepatocellular carcinoma, OS = overall survival.

We did a prognosis analysis based on the expression levels of G6PC versus gender, race, and pathological stages of patients with HCC. The results showed that there were significant differences in the expression of G6PC between male (log rank *P* = 2.2e-07, Fig. 6A) and female (log rank *P* = .35, Fig. 6B) patients. No significant differences in G6PC expression were found between Asian (log rank *P* = 6.6e-05, Fig. 6C) and White (log rank *P* = .015, Fig. 6D) HCC patients. There were no significant differences in G6PC expression between stage 2 to 3 (log rank *P* = .0038, Fig. 6E) and stage 3 to 4 (log rank *P* = .01, Fig. 6F) HCC patients as well.

### 3.5. G6PC expression correlates with immune infiltration in HCC

**Figure 7.**
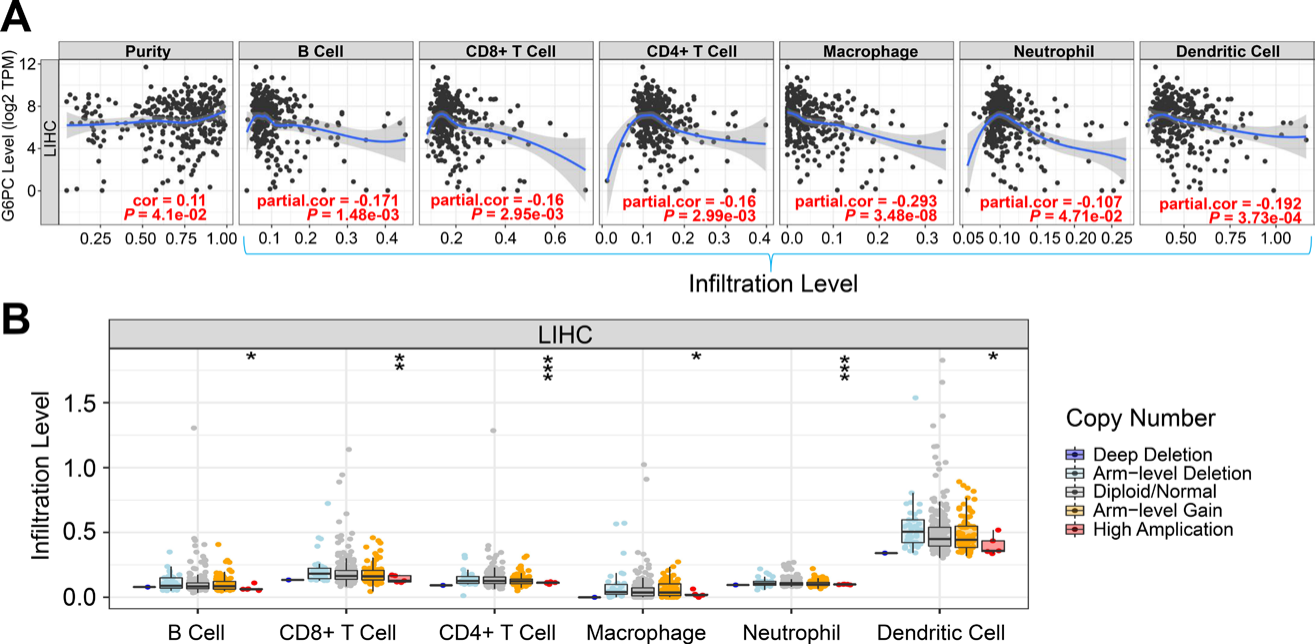
Correlations of GPC expression with infiltration of immune cells in LIHC. (A) G6PC expression had a positive relationship with tumor purity and a negative relationship with infiltration of immune cells such as B cells, CD4^+^ T cells, CD8^+^ T cells, macrophages, neutrophils and dentritic cells. (B) High amplication of G6PC had strong correlation with infiltration of immune cells. The box chart showed the distribution of each immune subgroup in each copy number state of LIHC (^*^P < .05, ^**^P < .01, ^***^P < .001). Abbreviations: G6PC = glucose-6-phosphatase catalytic, LIHC = liver hepatocellular carcinoma.

As shown in Figure 7A, G6PC expression level was positively correlated with tumor purity (partial.cor = 0.11, *P* = 4.1e-02), while negatively correlated with infiltration of B cells (partial.cor = −0.171, *P* = 1.48e-03), CD8^+^ T cells (partial.cor = −0.16, *P* = 2.95e-03), CD4^+^ T cells (partial.cor = −0.16, *P* = 2.99e-03), macrophages (partial.cor = −0.293, *P* = 3.48e-08), neutrophils (partial.cor = −0.107, *P* = 4.71e-02) and dendritic cells (partial.cor = −0.192, *P* = 3.73e-04). SCNA module was used to examine the effect of different SCNAs of genes on immune cell infiltrations, it revealed that among the various SCNAs, the copy number amplification was positively correlated with immune cell infiltration – the higher of copy number amplification, the stronger infiltration of B cells (^*^P < .05), CD8^+^ T cells (^**^P < .01), CD4^+^ T cells (^***^P < .001), macrophages (^*^P < .05), neutrophils (^***^P < .001), and dendritic cells (^*^P < .05, Fig. 7B).

## 4. Discussion

HCC is one of the most common malignant tumors in the world, ranking second in mortality and numerous gene networks and multiple signal transduction pathways are dysregulated in HCC.^[37,38]^ The rapid development of modern biotechnology and high-throughput sequencing technologies help people explore the mechanism of HCC pathogenesis at the molecular level, so as to further explore the occurrence and development process of HCC. However, the understanding of the biological mechanism of HCC is limited, and most of these studies are focused on a single dataset. Although HCC has different genomic characteristics, discovering the commonalities between heterogeneous genomic profiles is crucial for us to understand the development and progress of HCC. Therefore, the purpose of this study was to investigate the genes that were in common change in four HCC microarray datasets.^[38]^ In this study, computational bioinformatics analysis was used to systematically analyze the microarray datasets between HCC and adjacent tissues. By computational analysis of the differences in genome-wide gene expression between HCC tissues and para-tumor normal tissues, a total of 337 DEGs were identified, among which 126 genes were upregulated and 211 genes were down-regulated. GO pathways of DEGs were mainly enriched in cell division, condensed chromosome kinetochore and oxidoreductase activity, acting on paired donors, with incorporation or reduction of molecular oxygen. MCODE modular analysis of DEGs authenticated 8 key genes and the expression levels of CKS2 and G6PC impacted the survival of HCC patients with statistical significance.

CKS2, officially called cyclin-dependent kinase regulatory subunit 2, also known as cell cycle regulator, is elevated in HCC.^[39]^ CKS2 has been shown to be closely related to the progression of a variety of cancers. For example, studies have shown that expression of CKS2 is associated with poor survival in patients with tongue squamous cell carcinoma and have found that the CKS2 gene may have the potential as a biomarker for predicting the progression of superficial bladder cancer to muscle invasive cancer.^[40,41]^ CKS2 has been confirmed as an unfavorable prognostic marker in HCC.^[36,39,42]^ Previous reports demonstrate that overexpression of CKS2 may facilitate proliferation of HCC cells by reducing PTEN expression.^[42]^ High expression of EGFL7 can regulate HCC cell proliferation and apoptosis through CKS2-mediated Wnt/β-catenin signaling.^[43]^

The G6PC gene family consists of three members: G6PC, G6PC2 and G6PC3. These genes have different tissue-specific expression patterns, and mutations in all three genes have been linked to different diseases in humans.^[44]^ G6PC, also called Glucose-6-phosphatase catalytic subunit, multiple studies have shown that its high expression was significantly correlated with short-term recurrence and poor prognosis of ovarian cancer, and was expected to be one of the predictors of prognosis and recurrence of ovarian cancer.^[45]^ Here, we found that G6PC was down-regulated in a variety of tumors including HCC and KIRC, and low G6PC expression correlated with an unfavorable prognosis in HCC and KIRC.^[35,46]^ Chen et al have pointed out that G6PC can act as an independent prognostic factor, therefore, we then focused on exploring the association between G6PC and HCC. It has been shown that the expression of G6PC, which encodes the key gluconeogenesis enzyme glucogen-6-phosphatase, was significantly reduced in HCC, which was consistent with our research.^[47]^ In our study, G6PC expression negatively correlated with the OS in HCC patients. Moreover, G6PC expression predicts HCC patients OS independent on race and stage, but gender. Besides, G6PC expression is significantly positively related to tumor purity. The reason is that Figure 5A showed that the expression level of G6PC in hepatoma cells is lower than that in normal hepatocytes. However, the purpose here is to illustrate the correlation of G6PC expression with immune infiltration level. Tumor purity is a major confounding factor in this analysis, since most immune cell types are negatively correlated with tumor purity. In the database of Human Protein Atlas, we found that G6PC is mainly expressed in hepatocytes, proximal enterocytes and renal tubular epithelial cells, while immune cells do not express G6PC protein under normal circumstances. Therefore, the higher the tumor purity, the higher the expression level of G6PC and the lower the level of immune infiltration. TIMER analysis also showed a negative correlation between G6PC expression and infiltration of immune cells within the tumor, such as infiltrating B cells, CD8^+^ T cells, CD4^+^ T cells, macrophages, neutrophils and dendritic cells. The result suggests that G6PC may be involved in regulating the infiltration of immune cells in tumor microenvironment. G6PC encodes the key enzyme glucose-6-phosphatase, which functions in catalyzing glucose-6-phosphate into glucose during gluconeogenesis, thus its reduced expression resulting in decreased gluconeogenesis and reduced level of glucose as well, which may eventually affect tumor cell proliferation and tumor progression.^[47]^ Thus, G6PC may be used as a potential prognostic marker for HCC.

In summary, this study clarified the expression of G6PC in a variety of tumors, and its low expression was associated with poor prognosis of HCC, which may be used as a new potential prognostic marker and a potential molecular target for targeted therapy of HCC. However, this study had some limitations. First, clinical data from GEO did not apply to every sample. In addition, the microarray data were from different stages of liver cancer, and the expression levels of certain genes may not be exactly the same at different stages. Finally, the specific molecular mechanisms and biological functions of these potential candidate genes remained to be verified by further experimental studies, which could be extended to the interpretation of a variety of omics data including transcriptome, proteome, non-coding RNA, epigenome, metabolome and biological system level.

## 5. Conclusions

In this study, bioinformatics analysis of microarray data and literature review of HCC showed that there were interactions between core genes. G6PC may be a potential biomarker for the diagnosis and prognosis of HCC, which will be conducive to an in-depth understanding of the molecular mechanism of the development of HCC.

## Author contributions

Conceptualization: Li Tian and Yong Liao.

Formal analysis: Li Tian.

Investigation: Li Tian.

Methodology: Li Tian and Yong Liao.

Project administration: Yong Liao.

Software: Li Tian.

Supervision: Yong Liao.

Validation: Li Tian.

Visualization: Yong Liao.

Writing – original draft: Li Tian.

Writing – review & editing: Yong Liao and Li Tian.
